# Editorial: The subcellular architecture of mitochondria in driving cellular processes

**DOI:** 10.3389/fcell.2022.1003779

**Published:** 2022-08-26

**Authors:** Brian Cunniff, Jyoti K. Jaiswal

**Affiliations:** ^1^ Department of Pathology and Laboratory Medicine, the Robert Larner M.D. College of Medicine, University of Vermont, University of Vermont Cancer Center, Burlington, VT, United States; ^2^ Children’s National Research Institute, Center for Genetic Medicine Research, Washington, DC, United States; ^3^ Department of Genomics and Precision Medicine, George Washington University School of Medicine and Health Sciences, Washington, DC, United States

**Keywords:** mitochondia, mitochondrial dynamics, cell signaling, DRP1, OPA1, cell migration

Mitochondria regulate numerous cellular processes including metabolism, gene expression, motility and migration, as well as cell division, repair and death. Due to their ability to dynamically remodel, distribute throughout the cell, and interact with other organelles, mitochondria can perform its functions by acting locally or globally. The field of mitochondrial dynamics has thus grown from simple textbook observations of a static “kidney-bean” like energy producing structures to an organelle that works by dynamically changing its architecture and association with other organelles. The importance of the dynamic mitochondrial network and how integral it is to cellular function is highlighted by a growing list of diseases associate with defects in this process (Yapa et al., 2021). This Research Topic presents a collection of Original Research and Review articles that describe the machinery regulating mitochondrial dynamics, mitochondria-organelle interactions, and their relationship with cell-type specific functions (Yu et al., 2021; Cartes-Saavedra et al., 2022; Willingham et al., 2021; Bonjour et al., 2022), and examine how these regulate physiological and pathological processes such as cell division, cell motility, and tumor metastasis (Pangou and Sumara 2021; Madan et al., 2022; Boulton and Caino. 2022).

Mitochondrial fission is controlled through recruitment of the primary mitochondrial fission protein Dynamin-Related Protein 1 (DRP1) to mitochondria by adaptor proteins (Giacomello et al., 2020). In their research article, Yu and others clarify the control of adaptor protein-DRP1 interaction in regulating mitochondrial fission. They show that DRP1 spontaneously exists in multiple oligomeric states and the adaptor proteins show differential binding to these oligomers. While higher order oligomers are preferentially bound by Mitochondrial Fission Factor (MFF), adapter proteins MiD51/MiD49 can bind to a wider range of DRP1 assemblies. Their work shows that while MFF only recruits active DRP1, loss of MiD51/MiD49 impairs DRP1/MFF interactions. Therefore, although preferential recruitment of DRP1 by MFF occurs, the additional adapter proteins play a significant role in mediating this interaction (Yu et al.). These data identify selective binding dynamics of DRP1 adapter proteins to explain their non-overlapping roles in DRP1 recruitment to mitochondria in healthy cells that gets disrupted in disease caused by the loss of adaptor proteins (Koirala et al., 2013; Horn et al., 2020).

Unlike DRP1-mediated outer mitochondrial membrane (OMM) fission, Optic atrophy-1 (OPA1) regulates inner mitochondrial membrane (IMM) dynamics, mutations in which leads to dominantly inherited optic neuropathy that causes vision loss (Alexander et al., 2000; Olichon et al., 2006; Finkel et al., 2015; Weisschuh et al., 2022). Both OMM and IMM dynamics are controlled by, and in turn control, many inputs including calcium (Alexander et al., 2000; Horn et al., 2020). The research article by Cartes-Saavedra and others shows that cells lacking OPA1 have closer ER-mitochondrial contacts, which results in increased Ca^2+^ mobilization from ER to mitochondria. This was supported in cells from patients carrying OPA1 GTPase or GED domain mutations. Their study substantiates the role of OPA1 in modulating ER-mitochondrial coupling and shows even when mutant and WT OPA1 proteins are present together in patient cells, ER-mitochondrial Ca^2+^ homeostasis is disrupted, contributing to autosomal dominant optic atrophy (Cartes-Saavedra et al.).

Complimenting the mechanistic studies of mitochondrial shape change, Bonjour et al. examined how the mitochondrial ultrastructure changes during mouse eosinophil maturation and in inflammatory disease. They performed high-resolution mitochondrial ultrastructure analysis using transmission electron microscopy (TEM) and 3D tomography. This uncovered a reduction in mitochondrial area by 70% during eosinophil development that was attributed to increases mitophagy in immature eosinophils. They show that mitochondria form extensive contacts with other mitochondria and cellular organelles at a higher frequency in immature versus mature eosinophils. During asthma-induced inflammation, mitochondrial cristae remodeling occurs and mitochondrial contacts with granules increases. This highlights cell-specific mitochondrial ultrastructure remodeling that is responsive to increased inflammation, expanding the growing body of work highlighting mitochondrial ultrastructural changes in immune cell activity.

Expanding on the Original Research, Review Articles included in this Research Topic provide a comprehensive overview of contributions of mitochondrial dynamics to various physiological functions. In their minireview, Willingham et al. review subcellular specialization of mitochondrial structure and function in skeletal muscle and how plasticity of these mitochondrial features regulates function and physiological adaptations of skeletal muscle (Willingham et al., 2021). The review by Pangou and Sumara explore the connection between mitosis and cell division by discussing the regulation of mitochondria by mitotic machinery and how in turn mitochondrial function and inheritance regulates mitosis. They discuss the mitochondrial dynamics during mitosis and how its dysregulation is linked to diseases (Pangou and Sumara 2022). The discussion of mitochondrial involvement in cell division is also covered by Madan et al., who examine this for polarized epithelium and how mitochondrial dynamics and metabolism play a role during epithelial-mesenchymal transition and cell migration for wound healing, inflammatory responses and tissue remodeling (Madan et al., 2022). Extending this discussion to the pathological condition of tumor metastasis, Boulton and Caino have explored how the molecular mechanisms of mitochondrial dynamics affect the growth, metabolism, and invasiveness of tumor cells. They discuss how mitochondrial fission machinery regulates growth factor and ROS signaling that drive metastasis through regulation of tumor cell and its microenvironment (Boulton and Caino. 2022).

Together the primary research articles and reviews collected in this Research Topic highlight how control over the mitochondrial spatial, temporal and structural changes drive cell-type specific signaling, to support cellular function in healthy and diseased states. We thank the authors for their contributions and expert insights, the collection of which in this Research Topic will add new insights into the growing recognition of mitochondrial dynamics and its subcellular architecture in driving diverse physiological processes.
